# Biofeedback-Assisted Resilience Training for Traumatic and Operational Stress: Preliminary Analysis of a Self-Delivered Digital Health Methodology

**DOI:** 10.2196/12590

**Published:** 2019-09-06

**Authors:** Paul N Kizakevich, Randall P Eckhoff, Gregory F Lewis, Maria I Davila, Laurel L Hourani, Rebecca Watkins, Belinda Weimer, Tracy Wills, Jessica K Morgan, Tim Morgan, Sreelatha Meleth, Amanda Lewis, Michelle C Krzyzanowski, Derek Ramirez, Matthew Boyce, Stephen D Litavecz, Marian E Lane, Laura B Strange

**Affiliations:** 1 Bioinformatice Program Research Computing Division RTI International Research Triangle Park, NC United States; 2 RTI International Research Triangle Park, NC United States; 3 Kinsey Institute Indiana Unniversity Bloomington, IN United States; 4 Department of Psychiatry University of North Carolina at Chapel Hill Chapel Hill, NC United States; 5 Consultant Hilton Head, SC United States

**Keywords:** resilience, psychological, heart rate variability, Personal Health Informatics and Intervention Toolkit, PHIT, respiratory sinus arrhythmia, stress, psychological, relaxation therapy, biofeedback, psychology, well-being, mindfulness, digital health, mhealth

## Abstract

**Background:**

Psychological resilience is critical to minimize the health effects of traumatic events. Trauma may induce a chronic state of hyperarousal, resulting in problems such as anxiety, insomnia, or posttraumatic stress disorder. Mind-body practices, such as relaxation breathing and mindfulness meditation, help to reduce arousal and may reduce the likelihood of such psychological distress. To better understand resilience-building practices, we are conducting the Biofeedback-Assisted Resilience Training (BART) study to evaluate whether the practice of slow, paced breathing with or without heart rate variability biofeedback can be effectively learned via a smartphone app to enhance psychological resilience.

**Objective:**

Our objective was to conduct a limited, interim review of user interactions and study data on use of the BART resilience training app and demonstrate analyses of real-time sensor-streaming data.

**Methods:**

We developed the BART app to provide paced breathing resilience training, with or without heart rate variability biofeedback, via a self-managed 6-week protocol. The app receives streaming data from a Bluetooth-linked heart rate sensor and displays heart rate variability biofeedback to indicate movement between calmer and stressful states. To evaluate the app, a population of military personnel, veterans, and civilian first responders used the app for 6 weeks of resilience training. We analyzed app usage and heart rate variability measures during rest, cognitive stress, and paced breathing. Currently released for the BART research study, the BART app is being used to collect self-reported survey and heart rate sensor data for comparative evaluation of paced breathing relaxation training with and without heart rate variability biofeedback.

**Results:**

To date, we have analyzed the results of 328 participants who began using the BART app for 6 weeks of stress relaxation training via a self-managed protocol. Of these, 207 (63.1%) followed the app-directed procedures and completed the training regimen. Our review of adherence to protocol and app-calculated heart rate variability measures indicated that the BART app acquired high-quality data for evaluating self-managed stress relaxation training programs.

**Conclusions:**

The BART app acquired high-quality data for studying changes in psychophysiological stress according to mind-body activity states, including conditions of rest, cognitive stress, and slow, paced breathing.

## Introduction

### Background

Psychological resilience—the ability to recover from a traumatic experience and return to mental well-being—is critical to minimize health effects, such as anxiety, substance abuse, sleep problems, or posttraumatic stress disorder (PTSD) [1-6]. Exposure to trauma may leave the autonomic system in a chronic state of hyperarousal [7]. Heart rate variability (HRV), a measure of beat-to-beat cardiac interval variation, reflects vagal parasympathetic tone and changes in autonomic status [8]. Studies have found an association between PTSD and reduced HRV thought to be related to sustained hyperarousal and anxiety [9-14]. Conversely, higher HRV indicates greater flexibility and ability to regulate emotional responses, linking stress response to both enhanced mental health and resilience.

Reduction of arousal during or shortly after trauma exposure may prevent or reduce the likelihood of psychological distress, including PTSD symptoms [15-17]. Mindfulness meditation and relaxation training have been associated with a reduction in hyperarousal [17], may increase HRV [18,19], and hold promise for PTSD treatment [18,20]. HRV biofeedback, providing real-time HRV monitoring during relaxation training, has been shown to improve depression, anxiety, PTSD, and stress symptoms [21]. When practiced consistently, HRV biofeedback can also increase HRV and may help alleviate PTSD symptoms [22,23]; however, others have reported mixed results [24,25], indicating the need for further research.

### Objective

The Biofeedback-Assisted Resilience Training (BART) study is evaluating whether routine practice of slow, paced breathing with and without HRV biofeedback can enhance psychological resilience by facilitating an HRV rebound after a stressor task. To support the study, we developed the BART mobile app, enabling participants to practice relaxation training outside of a formal training environment. This paper describes the BART resilience training app, demonstrates HRV biofeedback, presents processes that may have wider applicability for mobile health research, and reports the interim results of app usage.

## Methods

### Study Population

The BART study is being conducted in a mixed population of military personnel, veterans, and civilian first responders at multiple sites across multiple states in the United States. We recruited participants from a convenience sample of Navy, Marine Corps, and Army Reserve units and National Guard armories from North Carolina, Georgia, and Virginia, and fire and police units in the Raleigh-Durham, North Carolina, area who volunteered to participate for a 60- to 90-minute onsite training session, practice their training at home, and complete a suite of survey assessments over the course of 1 year. Eligibility criteria included having a smartphone and knowing their password. We offered monetary incentives in addition to allowing them to keep their study-related heart rate (HR) monitor chest strap.

This study was approved by the University of North Carolina Institutional Review Board under an authorization agreement with the RTI International Committee for the Protection of Human Subjects; and the US Army Medical Research and Materiel Command, Office of Research Protections, Human Research Protection Office.

### Study and App Design

#### Study Protocol

The BART study is comparing 4 resilience training regimens: paced breathing at 5 or 6 breaths per minute, each with or without HRV biofeedback. Participants are randomly assigned to 1 of these 4 regimens and asked to practice paced breathing at least 3 times a week for 6 weeks, and thereafter for 12 months (Table 1). Participants use a chest-belt HR sensor to acquire HRV measurements, including participants randomly allocated to no HRV biofeedback. While such participants cannot observe changes in HRV during training, continuous acquisition of HRV data in the background enables posttraining analyses of physiological responses to cognitive stress and paced breathing training.

The study begins with a setup day (day 0) on which individuals provide their consent to participate, install the app on their smartphone, complete baseline assessments, learn to use their HR monitor (Polar H7; Polar Electro, Bethpage, NY, USA), and practice 1 resilience training session, which includes a cognitive stress game. Special consideration is given to wearing the Polar HR sensor, linking the sensor to the BART app, and acquiring good-quality HR measurements. After this initial setup and instruction, participants execute all activities on their own for the duration of the study under the scheduling and direction of the BART app.

**Table 1:** Schedule of participant activities across the yearlong study duration.

Time	Resilience training	Resilience training with cognitive stress	Assessment	Incentive (US $)
Day 0	N/A^a^	Practice once	Baseline part 1	15
Days 0-3	N/A	N/A	Baseline part 2	5
Days 0-3	N/A	N/A	Baseline part 3	5
Weeks 1-6	Practice twice/week	Practice once/week	Weekly survey	10/week
Months 3, 6, and 9	Practice twice/week	Practice once/quarter	Quarterly survey	20/quarter
Month 12	Practice twice	Practice once	Final survey	20

^a^Not applicable.

BART app design was governed by the study protocol and schedule of participant activities (Table 1), the prescribed resilience training and stressor regimens, the mechanisms for helping participants to complete the study activities, and incentives to encourage adherence. A suite of self-report measures (eg, anxiety, posttraumatic stress, sleep quality, resilience) are taken at baseline, with a subset taken quarterly and at 12 months. Scheduling of activities is provided via the app, along with incentives to support adherence over the initial 6 weeks and throughout the 12 months. Owing to the geographic distribution of study recruitment sites, participants enter the study incrementally, thereby allowing a small study team to recruit, take consent, and provide initial training at various locations over an extended period. Consequently, each participant’s protocol schedule is based on their personal study entry date.

#### App Development

Our previous work in predeployment stress inoculation training, HRV biofeedback, and mobile technologies for mindfulness-based stress reduction strongly influenced our design of the BART app [18,25,26]. Each of these studies involved stress relaxation training, a cognitive stressor, and HRV assessments. We reviewed our lessons learned from these studies to refine processes and incorporate new sensors and mobile technology in the BART app. Smartphone-delivered health and HRV biofeedback analyses of the prior Personal Health Intervention Toolkit for Duty research app [27] constituted the foundation for app development.

We implemented the BART app using the Personal Health Informatics and Intervention Toolkit (PHIT), a development framework geared to research-oriented mobile apps [28-30]. The PHIT framework eases app development for acquiring data, including self-report instruments, ecological momentary assessment diaries, cognitive tests, and game-like activities. For sensor data collection, PHIT supports intrinsic (eg, global positioning system, motion) and Bluetooth 4.0 data streams (eg, HR monitors). All data are tagged with study protocol, participant, date and time stamps, and other contextual information, then encrypted and stored locally in the app space. A virtual advisor provides a logic layer where analysis and planning take place. An activity manager schedules self-report and sensor data collection, intervention and training, alerts, incentive feedback, and behavior change according to the study protocol.

PHIT modules are implemented using XML and employ PHITScript to construct program logic and activate special app functions, such as collecting sensor data or scheduling notifications. Apps using the PHIT framework run locally without the need for an active internet connection. PHIT is based on Apache Flex (Apache Software Foundation) and AIR (Adobe Systems) technologies, which are both open source and widely used for mobile game development. A requirement for the BART study was that participants would use their own smartphones or tablets, necessitating app compatibility with both Android and iOS devices as provided by Adobe AIR.

All acquired and derived data are stored on the device in an encrypted SQLite (SQLite Consortium) database and periodically uploaded by the participant to a secure central data server. To eliminate financial burden, neither continuous internet access nor use of the participant’s cellular data plan is required. Rather, data are uploaded whenever Wi-Fi internet access is available, and at the participant’s direction and convenience, either via Wi-Fi or the participant’s cellular data plan.

#### App Architecture

**Figure 1:**
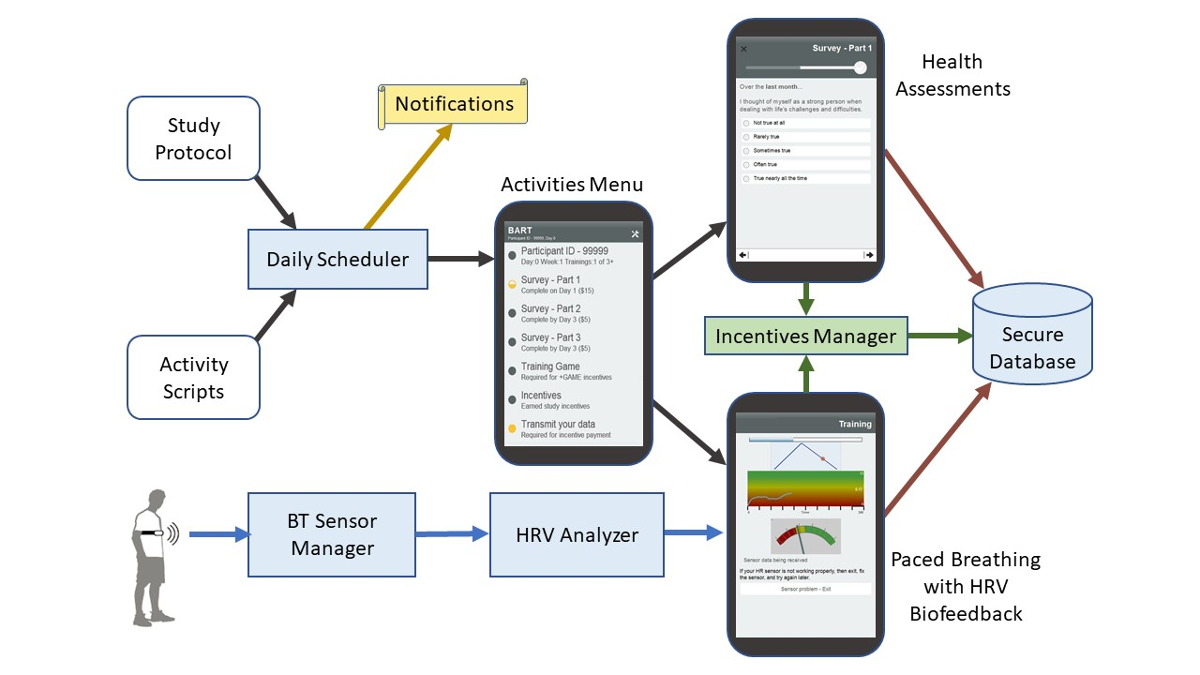
Overall architecture and major components of the Biofeedback-Assisted Resilience Training (BART) study mobile app. BT: Bluetooth; HRV: heart rate variability.

**Figure 2:**
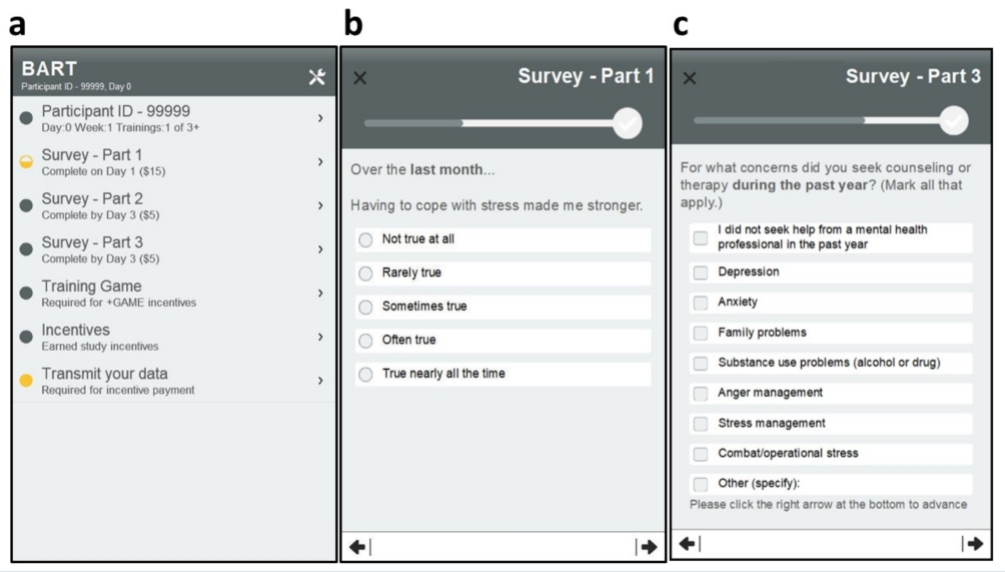
Biofeedback-Assisted Resilience Training (BART) app home screen activities menu and examples of health assessment survey questions. (a) Activities menu; (b,c) sample survey questions.

The BART operational schema (Figure 1) centers on a participant activities menu with various tasks such as health assessments, resilience training, and data uploads. The activities menu (Figure 1 and Figure 2a) is updated daily by a schedule manager according to protocol specifications and personal progress, using labeling and icon references as defined by PHITScript programming. Each day, activities are listed or removed, and a local notification is posted to the participant as a reminder to complete their activities. The menu may also be updated by changing the icon to note an incomplete activity, removing a completed activity from the menu, or tagging a listed activity with key user information—for example, advising on the number of resilience trainings remaining to meet incentive payment requirements for the current study week.

Primary outcome measures acquired via the app are resilience (Connor-Davidson Resilience Scale), coping measures (Brief Coping Scale, Perceived Stress Scale, and Posttraumatic Growth Inventory), and sleep problems (Sleep Disturbance Scale). Secondary outcomes are mental health (measured by the PTSD Checklist, 7-item Generalized Anxiety Disorder scale, and Center for Epidemiologic Studies Depression Scale), physical health (Short Form Health Survey), and alcohol use problems (Alcohol Use Disorders Identification Test). Covariates are combat and deployment, recent tobacco and caffeinated beverage use, age, education, use of other relaxation techniques, and interest in learning relaxation skills. These measures, along with demographic information, were aggregated into a set of brief survey instruments to be completed at baseline (surveys 1-3), weekly, quarterly, and at 12 months.

Health assessments are administered via brief self-report instruments, typically with a single question per screen (Figure 2b and c). As the user advances through each assessment, a graphic indicator informs progress toward completion. At completion of each self-report measure or resilience practice exercise, an incentives manager records the earned incentive to the database (Figure 1). The user is then advised to upload data or defer the upload to a more convenient time. The activities menu may also be updated. For activities with HR sensor data streams, a Bluetooth interface manager links the sensor and receives beat-by-beat HR information for HRV analysis. Once initiated, this process executes autonomously in the background while the participant performs resilience training. The raw HR and derived HRV measures are provided for feedback display and saved in the app database.

#### Heart Rate Variability

The BART project employs real-time HRV analysis to provide physiological biofeedback during resilience training. Beat-by-beat heart intervals, also called interbeat intervals, are acquired continuously during each training session from a Bluetooth Low Energy (Bluetooth Special Interest Group) HR monitor. The raw interbeat intervals are streamed in real time to an HRV analysis module and stored in the app database to allow for subsequent offline quality review and analysis.

Three variants of HRV measures are determined using the Porges-Bohrer HRV analysis methodology [31,32]: respiratory sinus arrhythmia (RSA), low-frequency HRV, and wideband HRV. RSA reflects parasympathetic vagal activity for expected spontaneous breathing rates, whereas low-frequency HRV is thought to reflect sympathetic activity, as well as other cardiovascular regulatory systems. The wideband measure ensures that very low breathing rates during paced breathing exercises are properly measured. Multimedia Appendix 1 provides HRV data processing details.

#### Resilience Training

**Figure 3:**
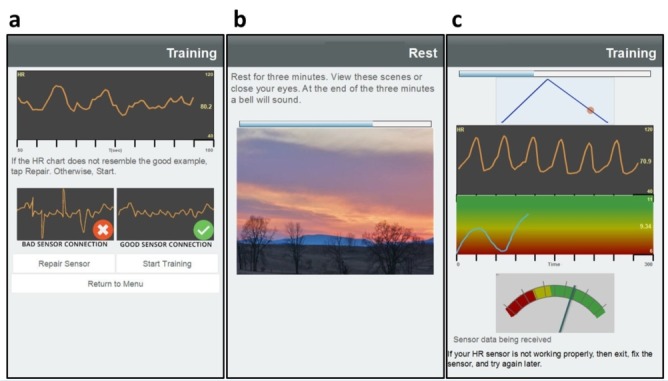
(a) Resilience training begins with verification of heart rate (HR) data quality to ensure high-quality heart rate variability (HRV) biofeedback. (b) Once the HR is checked, participants sit at rest for a 3-minute baseline period. (c) Then participants receive paced breathing via an audiovisual graphic animation (top), while the HR signal, HRV trend, and instantaneous HRV meter are displayed in real time (bottom).

We asked participants to practice resilience training 3 times each week for 6 weeks, a 2-step process comprising a 3-minute resting segment and a 5-minute resilience training segment. Each participant was randomly allocated after consent to receive 1 of 4 resilience training regimens: paced breathing at 5 or 6 breaths per minute, with or without HRV biofeedback. Before training, the participant is asked to be in a quiet place and put on the Polar H7 HR monitor. When the participant is ready, the HR monitor is activated and a beat-by-beat HR trend is displayed to check signal quality (Figure 3a). The participant reviews the HR trend and decides whether to proceed to resilience training or take measures, such as adjusting or moistening the chest strap sensor, to improve data quality. Resilience training begins with a 3-minute resting segment to relax the participant and establish baseline HRV measures. During this time, the participants may close their eyes or lightly focus on a series of peaceful landscapes that fade from one to another at 30 second intervals (Figure 3b). A narrator announces when each minute arrives to help reduce anxiety owing to waiting for the resting segment to finish.

For participants receiving resilience training *without biofeedback*, an animated ball is displayed as rising and falling upon a triangular graphic for paced breathing resilience training (Figure 3c top). Participants inhale as the ball rises and exhale while the ball falls, with ball movement set at 5 or 6 breaths per minute, with an inspiration to expiration ratio of 0.435 and an end-inspiration and end-expiration pause of 1.5 seconds. An audible tone with rising and falling pitch is played in synchrony with the rising and falling ball to allow for paced breathing with eyes closed.

For participants receiving resilience training *with biofeedback*, the animated ball and audible tones are rendered in similar fashion to that without biofeedback. Two modes of graphic biofeedback are provided: a trending HRV chart and a real-time dynamic HRV meter. The chart and meter are updated every 2 seconds against a color-coded background to show movement between calm (green) and stressful (red) psychophysiological states, reflecting higher and lower parasympathetic activation.

On study days 0 and 1, and after 6 weeks of training, participants complete an enhanced training regimen called the Training Game. The Training Game builds on the basic resilience training exercise by incorporating the Eriksen flanker task [33], a game-like cognitive stress exercise designed to elicit psychophysiological stress. The Eriksen flanker task heightens psychological stress by requiring attention, providing anticipation, and imposing conflict in higher brain function. As before, HRV is measured throughout the Training Game, thereby allowing for objective assessment of resilience before and after 6 weeks of training.

**Figure 4:**
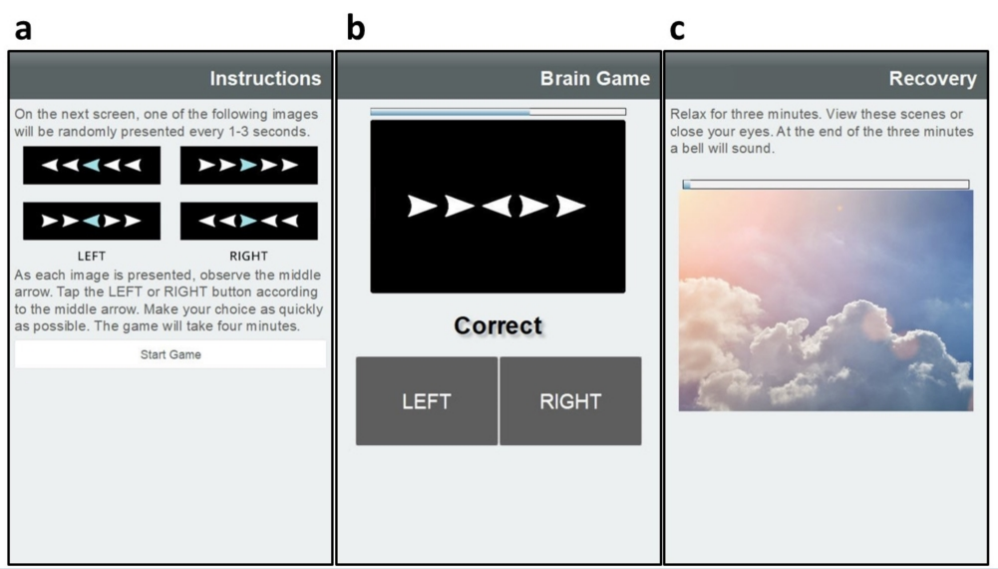
The cognitive Training Game stressor exercise is preceded by (a) an instruction screen, followed by (b) 4 minutes of Eriksen flanker stimuli with user response, and completed with (c) 3 minutes of poststress recovery.

The Training Game begins with a 3-minute rest, followed by instructions on performing the Eriksen flanker task (Figure 4a). When ready, the participant begins the Eriksen flanker task, which presents a series of stimulus screens comprising a field of arrows pointing to the left or right, with a central arrow that may be congruent or incongruent in direction with the 4 bounding arrows on either side (Figure 4b). The 4 bounding arrows are randomly rendered as pointing left or right, resulting in 4 available stimulus combinations. At a random interval ranging from 1 to 3 seconds, 1 of the 4 combinations of the central and flanking arrows is selected at random and presented for 400 milliseconds. Participants have 2.7 seconds to respond by tapping the left or right button to indicate the direction of the central arrow. The Eriksen flanker task continues presenting stimuli for 4 minutes, then the BART app advances to a 3-minute poststress recovery phase of sitting at rest (Figure 4c). After recovery, resilience training is provided as previously described with paced breathing at 5 or 6 breaths per minute, with or without HRV biofeedback.

#### Protocol Adherence and Incentive Management

**Figure 5:**
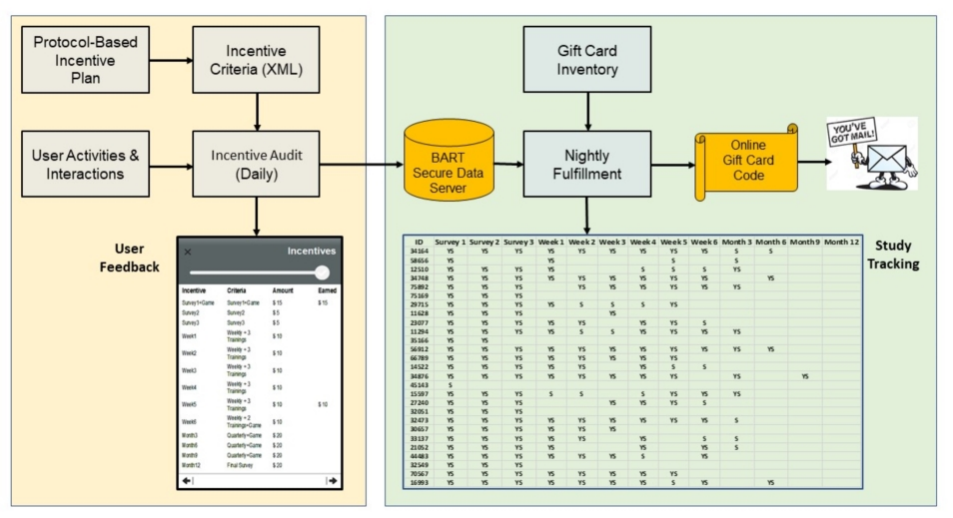
Incentive management data flow and processes conducted within the Biofeedback-Assisted Resilience Training (BART) app (left) and in the secure backend data server (right) for monitoring and rewarding participant adherence.

Adherence to study interventions and data collection is a common challenge in any research study, and especially when participants are asked to essentially perform the study on their own, albeit with app support. To support the frequency of resilience training (3 time per week), weekly assessments, and the yearlong period of follow-up assessments, the BART study design incorporated an incremental incentives approach to encourage participants to do scheduled activities and stay engaged across the duration. We were concerned, however, that manual monitoring of several hundred participants with time-shifted protocol schedules could be error prone and cause missed payments or awarding unearned payments. To mitigate such risk, we implemented incentive management to standardize both incentive qualification and automated distribution of incentive awards (Figure 5).

In concert with the BART protocol (Table 1), incentives are earned providing that the participant completes the requisite activities (Figure 5). Incentive criteria, along with labeling and monetary values, are coded in PHITScript and checked both daily and after the participant exits each activity module. Whenever the participant completes the criteria for a specific incentive, an incentive fulfillment request is stored to the local app database, and the in-app incentives table is updated to inform the user that an incentive has been earned and is pending award. When study data are transferred to the secure central data server, the incentive fulfillment request is noted for processing. Each night a procedure scans all participants for pending fulfillment requests, identifies unpaid incentives, emails a gift card code in the appropriate amount to participants, and tags the incentive payment as fulfilled.

To further support the incentive process, monitor payments, and validate the accuracy of the incentive management process, a report of incentives earned and paid is produced weekly so that study staff may check each participant’s incentive record. This report not only aids in confirming payments, but also helps to resolve any problems that may have been experienced by participants and supports monitoring adherence across all participants.

#### Privacy and Security

Ensuring privacy and security of data and on-device analysis results is an absolute necessity for ethical reasons, to meet human studies requirements, and to support data quality in the conduct of self-managed mobile research protocols. Participants are provided a randomized participant identification (ID), which uniquely links all acquired data to that individual without any personally identifiable information. They also enter a self-defined secret 4-digit personal identification number (PIN) to prevent access by other individuals. When in use, the app screen deactivates after a set period (eg, 2 minutes) of no interaction, and current data and activity is hidden. The 4-digit PIN must then be entered to unlock the screen and allow the participant to continue.

Implementing a study using an app installed on the participants’ devices requires app installation from a public app store. Since anyone might download and install the app, and possibly upload false data, we addressed this quality and security risk by including an app lockout requiring an unlock password. After participants consent to take part in the study, the unlock is revealed, the app installed, and the password entered to activate. This prevents extraneous persons from entering and corrupting the study.

All data are stored locally on the device in an encrypted SQLite database within the BART app, thereby permitting use without requiring a continuous internet connection. Data are stored using a 128-bit Advanced Encryption Standard algorithm with no personal identifying information. Data are periodically uploaded to a central secure data server whenever Wi-Fi internet access is available, thereby reducing use of the participants’ cellular data plans. Data are transferred using the secure https protocol and stored in a secure SQL server database, which is accessible only to authorized persons via user ID and password authentication.

#### Data Analysis

To showcase how the BART app is being used and to present examples of the HRV measures during resilience training, we conducted a limited, interim review of user interactions and study data. Consequently, data presented here do not address the study hypotheses on the effectiveness of various training modes on building resilience. Analysis of training effectiveness on resilience and other outcome measurements will be addressed separately after completion of data collection.

We based data regarding app usage on participant rostering records and earned incentives reported. For each study activity (Table 1), we tallied a completed measure—that is, the number of participants who completed the activity and earned the corresponding incentive. Since each participant has a unique study calendar based on individual starting date, the activity schedule differs across participants. We therefore tallied the number of participants who were scheduled to perform each activity adjusted according to their individual start date (ranging from June 2017 to September 2018) until this analysis on October 1, 2018. Finally, we determined the ratio of completed to scheduled activities as a compliance measure for each required study activity. We calculated these analyses using Excel 2019 (Microsoft Corporation).

We reviewed the psychophysiological stress response during the cognitive stress and biofeedback training using the wideband biofeedback HRV measure across all segments (rest, stressor, recovery, and training). Our analysis was restricted to data taken during the first week of participation, before substantive resilience practice would yield any training effect.

We computed descriptive statistics for the subpopulation extracted for the HRV measurement examples, with categorical variables reported by frequencies and numeric variables by mean (SE). We analyzed grouped HRV data using a univariate general linear model and present the data graphically as mean (2 SE). We used unpaired *t* tests to evaluate changes in HRV for sequential training segments. We conducted statistical analyses using IBM SPSS Statistics 25 (IBM Corporation).

## Results

### Resilience Training and Protocol Compliance

**Figure 6:**
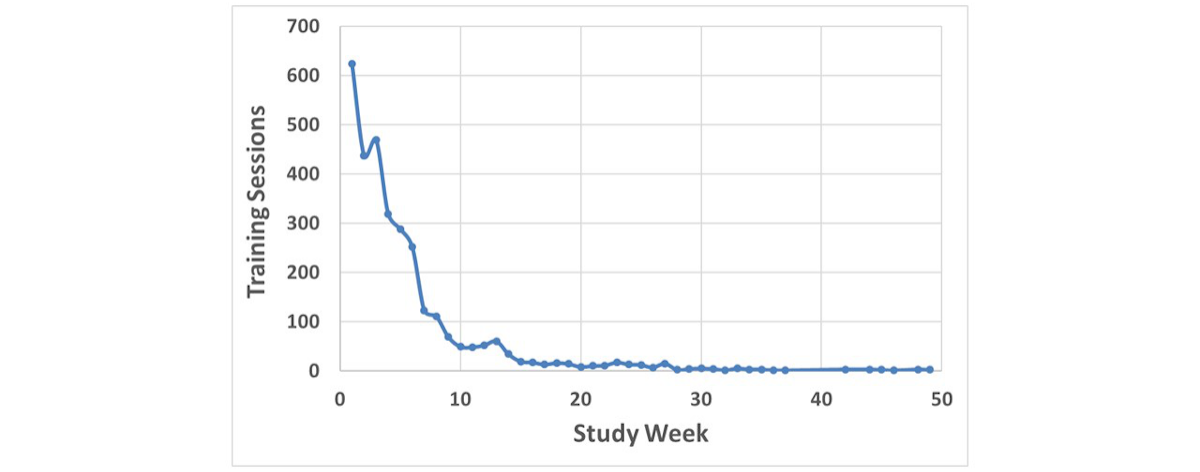
Total number of resilience training sessions across participants by study day.

Of the 328 enrolled participants to date, 207 (63.1%) adhered to the study training regimen of 3 resilience training sessions per week for at least six weeks. In total, 3136 training episodes had been performed in this subset across the first 180 days of each participant’s involvement (studyDay; Figure 6). At first, compliance with the training regimen was excellent, with over 600 sessions conducted during the first week by the 207 participants who completed the 6-week training regimen. However, over the next several weeks, training compliance fell by almost one-third, and later to about one-half after a month. Following the 6-week training period, compliance was diminished far below 3 trainings per week. However, a small number of participants continued resilience training for at least six months, with several continuing for nearly a year (not shown on the plot in Figure 6).

**Figure 7:**
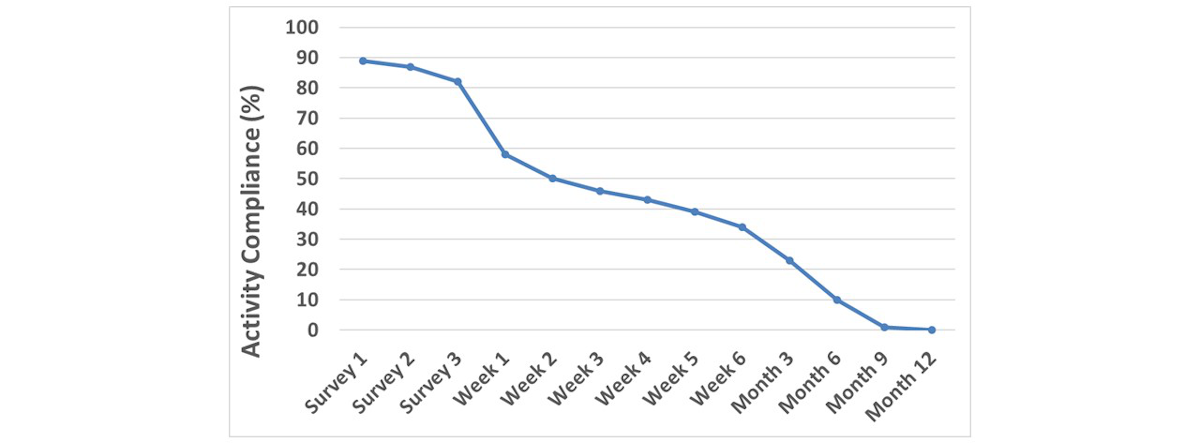
Compliance with scheduled study activities (listed in Table 1).

We also examined adherence to completing self-report health and wellness surveys at baseline, weekly for 6 weeks, and quarterly for up to 12 months according to the study protocol (Table 1). Among the 328 enrolled participants, compliance with completing scheduled surveys decreased across the study duration (Figure 7). Although participants were asked to complete surveys 1 to 3 immediately after installing the app, many were short on time and indicated that they would do them later that day. However, 11.0% (36/328) did not even complete the initial survey. Each week, and quarter, as each data collection survey was scheduled, participants were reminded to complete the pending survey and receive their incentive via a smartphone notification. A total of 1760 incentives were earned and automatically awarded from June 2017 through September 2018. Despite this support by the BART app, completion rates fell to 50.0% (164/328) by week 2, then to 22.9% (75/328) at 3 months and 10.1% (33/328) at 6 months.

### Heart Rate Variability

We included a subset of the dataset, comprising 49 men and women, aged 20 to 60 years (mean 36.7, SE 10.6), in the HRV review. Of the 49 participants in the subset, 23 (47%) were female and 26 (53%) were male. We excluded HRV values that were out of the expected range (HRV<0 or HRV>10) as outliers, as such data are likely due to interbeat interval artifacts. We included multiple HRV measures per individual segment, ranging from 305 to 613 measures.

**Figure 8:**
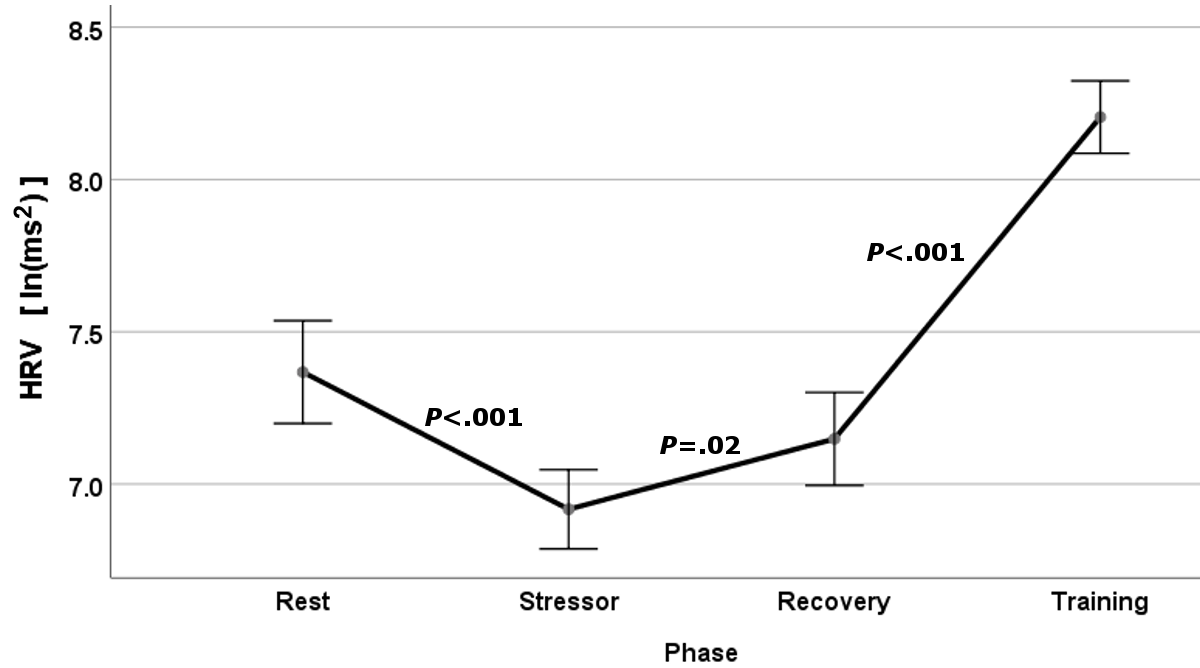
Heart rate variability (HRV) at rest and during cognitive stress, recovery, and paced breathing. Error bars represent ±2 SE.

We present grouped results for HRV, using the wideband biofeedback HRV measure, for each segment of the cognitive Training Game stressor exercise (Figure 8). As expected, HRV decreased from mean 7.37 (SE 1.77; n=305 measures) at rest to mean 6.92 (SE 1.41; n=515 measures) during the stressor phase, reflecting a reduction in parasympathetic activation during the Eriksen flanker stressor task (*P*<.001). Later during the poststress recovery segment, the HRV rebounded to mean 7.148 (SE 1.42; n=373 measures), approaching the prestress baseline (*P*=.02). During training, HRV increased very significantly to mean 8.205 (SE 1.39; n=613 measures), reflecting strong parasympathetic activation with slow paced breathing (*P*<.001).

## Discussion

### Principal Findings

A large variety of mobile apps for stress reduction [27,34,35], mindfulness training [36,37], biofeedback [38], and HRV measurement [39] have emerged over the last decade, both for general use and as adjuncts to specific disease interventions [38,40]. Most merely provide narrative training support and practice reminders, with little evidence of efficacy [36]. Furthermore, most app implementations do not have concomitant self-report or physiological data gathering as is necessary for evaluating efficacy. As the BART app is built on the PHIT mobile health research platform [28], we are able to acquire research data throughout the 6-week training regimen, tag data according to training segments (ie, rest, stress, paced breathing), and acquire physiological HRV measurements to support hypothesis testing in our primary BART evaluation study. We expect, therefore, that using our self-delivered digital health methodology will improve the understanding of the efficacy and utility of mobile, self-directed mind-body interventions.

Physiological biofeedback during paced breathing resilience training and objective assessment of psychological arousal would not be possible without real-time continuous monitoring of HRV by the BART app. The HRV results during the Training Game exercise for the rest, cognitive stressor, recovery, and training segments (Figure 8) are consistent with previous results found in our predeployment stress inoculation studies [25,26], where we observed a significant decrease in HRV (RSA) during cognitive stress and a significant increase during relaxation breathing. A similar reduction in HRV with cognitive stress has been reported by other investigators during mental arithmetic [41] and random number generation [42] tasks. By assessing the vagal-mediated RSA throughout each resilience training episode, we can readily observe changes in arousal due to different psychophysical states (eg, rest, stress). Therefore, any potential improvement in base arousal or resilience to (cognitive) stress after the 6 weeks of resilience training should be readily demonstrated.

A limitation in this study is the use of cognitive stress as a surrogate for combat and operational stressors in this military population. Risk of death, exposure to combat or casualties, disconnection from loved ones, and working in extreme and unusual environments are examples of trauma that our study population might experience. Such stressors cannot be readily mimicked, nor should they, as previously exposed participants could experience negative reactions to simulated exposures. Use of a controlled cognitive exercise provides an alternative, safe, and common context to assess stress reactivity for evaluation of relaxation training.

Along with the stated benefits, the BART study has yielded a variety of lessons for such self-directed app-based research. Using personal mobile apps not only to collect information, but also to manage protocol-based task scheduling, reminder notifications, and intervention activities, makes the study essentially self-administered by each participant. Unforeseen events, such as participant smartphone replacement, forgetting 4-digit security PINs, and assorted HR monitor failures, necessitated the implementation of technical support resources. We did this via website, telephone, and email interactions, with issue and resolution tracking using Jira Software v7.11.2 (Atlassian). Maintenance of personal interest, usability of sensors and devices, adherence to procedures, and timely technical support are critical in retaining participation for the study duration.

The nature of our study population (primarily military reserve units) imposed a requirement to recruit participants, then immediately install the BART app and provide initial training in a group setting, often with more than 20 individuals present. These large groups compromised our process to establish Bluetooth links between individual participant’s HR monitor and smartphone in a multiparticipant environment. We addressed this by having participants configure their app in small subgroups, which eased the installation and setup process considerably.

Furthermore, while the selected HR monitors work quite well with exercise, obtaining a good HR signal was often difficult while the participant is sitting at rest (ie, not sweating). Multiple adjustments of the sensor strap and repeated configuration attempts were often required, and we suppose that continued problems of this sort likely contributed to participant dropout. Advances in wearable HR sensor technology, such as upper arm photoplethysmography, may make them easier to use and more reliable under resting conditions than a device designed for exercise. As such devices emerge with enough accuracy for HRV measurement, we expect to improve protocol adherence and reduce participant dropout due to problems experienced with the HR sensor.

Incorporation and automation of incentive management is a vital aspect of the BART app. We are using monetary incentives, an important component of research projects, to support adherence to study procedures and reward participants for carrying out certain tasks, such as completing a survey or resilience training. Since participants have individual start dates, their individual calendar of study activities will differ across the study population, making manual monitoring of study adherence both time consuming and error prone. By embedding adherence management, we can check protocol activities frequently, then reward participants immediately using automatic, incremental incentive payments. Weekly reports to study staff on incentive payments yielded useful feedback on protocol adherence and the potential for intervention by study staff to help keep individual participants on track.

Retention of users is a common issue with mobile apps in general. Bonnie [43] reported that 90% of users stopped using apps within 30 days and 95% by 90 days after installation. In contrast, the BART app methodology of supporting participants with incentives and usage feedback allowed the study to retain over 20% of enrolled participants after 60 days and roughly 10% at 90 days.

While a key component of ensuring optimal study participation, automation of incentive management was not without issues. Initially we had a somewhat complex set of requirements for participant incentives, including requiring their resilience trainings plus completion of the weekly survey in each of the first 4 weeks to receive the incentive payment. Furthermore, participants were given 4 days to compete the survey, and then it was removed from the activities menu. Despite instructions via the embedded incentive requirements table, several participants complained that they did not receive incentives. Upon review, we found that they did not fully meet the requirements but, as they met most, we decided to award the incentives anyway. We then relaxed the requirements, while still asking that these tasks be completed (or at least initiated), so that such persons would not drop out of the study. Nonetheless, having the automated incentive checking and database recording was helpful to review these cases and to consider the participant’s actions and understanding how to better incentivize study activities.

### Conclusion

Results presented is this paper merely showcase features and capabilities of the BART app, along with preliminary data on app usage and demonstration of analyses of real-time sensor-streaming data, such as the psychophysiological HRV response to cognitive stress and paced breathing training.

Currently distributed for the BART research study, the BART app is being used to collect self-reported survey and HR sensor data for comparative evaluation of paced breathing relaxation training with and without HRV biofeedback. Our preliminary ad hoc analyses indicate that the app acquires high-quality data for studying changes in psychophysiological stress according to mind-body activity states, including relaxation and cognitive stress conditions. However, no conclusion of effectiveness, or noneffectiveness, of the biofeedback-assisted relaxation training intervention should be drawn from these data.

## Appendix

Multimedia Appendix 1 Heart rate variability methodology and validation.

## Abbreviations

BART: Biofeedback-Assisted Resilience Training
HR: heart rate
HRV: heart rate variability
ID: identification
PHIT: Personal Health Informatics and Intervention Toolkit
PIN: personal identification number
PTSD: posttraumatic stress disorder
RSA: respiratory sinus arrhythmia
