# Supplementary material for: Biofeedback-Assisted Resilience Training for Traumatic and Operational Stress: Preliminary Analysis of a Self-Delivered Digital Health Methodology
Source: JMIR Mhealth Uhealth. 2019 Sep 6;7(9):e12590. doi: 10.2196/12590 (PMC6754694; doi:10.2196/12590)
Supplement: Multimedia Appendix 1 [file mhealth_v7i9e12590_app1.pdf]

Appendix 1: Heart Rate Variability Methodology and Validation

The Personal Health Informatics (PHIT) technology employs real-time HRV analysis for assessing physiological arousal and biofeedback training [1,2]. Beat-by-beat heart intervals, also called interbeat intervals (IBIs), are acquired continuously (Figure 1) from a Bluetooth Low Energy heart rate monitor (HRM), Such as the Polar H7 or H10 with enough IBI timing accuracy for quality HRV measurement. The raw IBIs are streamed in real time to an HRV analysis module, while also stored in the app database to allow for subsequent offline quality review and analysis.

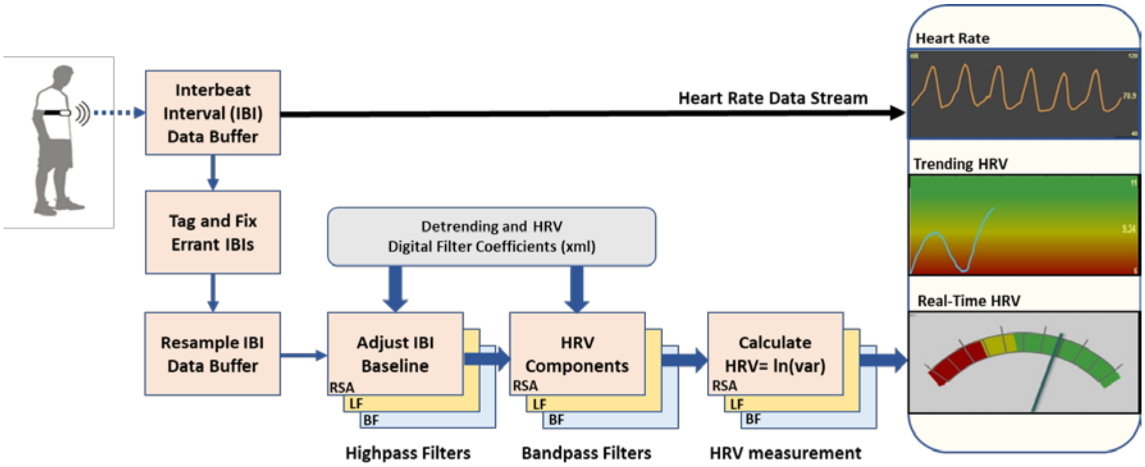

Figure 1. BART heart rate variability processing schema based on the Porges–Bohrer method.

This data stream is buffered to yield a series of short overlapping IBI epochs, each with a two-second delay from the start of the prior epoch. Each epoch is reviewed using an artifact detector [3] which identifies IBIs that are 35% longer or shorter than the previous beat, then replaces these errant beats with an IBI estimate from previous good beats. Time-domain filtering of the sequence generates a valid central section of data, the length of which varies as a function of the frequency response of the filter. The epoch duration is selected to allow for synchronous estimation from a valid 70-second segment of filtered data for all three HRV signals.

Three variants of HRV measures are determined: respiratory sinus arrythmia (RSA; 0.12 – 0.40 Hz), low-frequency HRV (LFHRV; 0.05 – 0.10 Hz), and wideband HRV (BFHRV; 0.05 – 0.40 Hz) for biofeedback training. RSA reflects parasympathetic vagal activity for expected spontaneous breathing rates, while LFHRV is thought to reflect sympathetic activity, as well as other cardiovascular regulatory systems. Normal respiration generally exceeds seven breaths/minute, so the prescribed paced breathing training at five and six breaths/minute would fall below the 0.12 Hz RSA filter cutoff. Consequently, a wideband BFHRV measure spanning the LFHRV and RSA frequency range was conceived to provide accurate HRV biofeedback during paced breathing resilience training.

As the time series of sequential heart periods have varying intervals, the IBI epochs were resampled to yield a uniform series at five Hz. This resampled series was processed with a cubic polynomial filter (zero mean, 3rd order) with a low-pass cutoff frequency of 0.095 Hz for RSA and .0495 Hz for the other HRV measures [4]. The filter was convolved with the resampled time series to create a template of the low frequency and aperiodic trend components in the time series. The trend component was subtracted from the re-sampled heart period time series to create a stationary zero mean time series that is statistically appropriate for filtering with a finite impulse response filter. The resulting time series has a periodic oscillation at the frequency of spontaneous respiration [5]. The removal of aperiodic trend and low frequency activity provides an opportunity to more accurately quantify RSA, which may be masked or distorted by slower activity and aperiodic trend.

A Chebychev type I bandpass filter was applied to the residual series to remove any variance outside the bandwidth of spontaneous respiration (0.12–0.40 Hz) for RSA and outside the given frequency bands for LFHRV and BFHRV. Given the roll-off of the filters used, variance associated with oscillations slightly outside the filter bandwidth will be partially passed through to the final filtered series. The variance of the central 20 seconds of the final filtered series is transformed with a natural logarithm ($\ln(ms^2)$), to generate the final estimates of HRV within each band. The logarithmic transformation reduces skewness and kurtosis of HRV parameters to enable the data to more closely conform to the assumptions of normality [6].

To validate the automated HRV analyses of the BART mobile app, the real time app-derived HRV measures were compared to established laboratory-based offline analyses algorithms for HRV analysis. Ninety-eight (98) subjects were recruited, including both 29% female and 71% male participants. Data were acquired during periods of rest, cognitive stress, post-stress recovery, and paced breathing biofeedback relaxation training. The linear regression between app-based and offline IBI measures were in convergence ($y = .98x + .63$) displaying a high correlation R^2 of .78. For BFHRV, while not in convergence ($y = .93\,x + .49$), a high correlation R^2 of .86 was observed. For the LFHRV parameter ($y = 1.08x + .63$) differs from the unity but also had a high correlation R^2 of .80. Finally, while RSA was in convergence ($y = 1.02x + .34$) had a moderate correlation R^2 of .67.

References

1. Eckhoff, RP, Kizakevich, PN, Bakalov, V, Zhang, Y, Bryant, SP, Hobbs, M. A personal health intervention toolkit for building mobile health applications. JMIR mHealth and uHealth, 2015;3(2):e46. PMID: 26033047.
2. Kizakevich, PN, Eckhoff, R, Brown, J, Tueller, SJ, Weimer, B, Bell, S, Weeks, A, Hourani, L, Spira, JL, King, LA. PHIT for Duty™, a Mobile Application for Stress Reduction, Sleep Improvement, and Alcohol Moderation. Mil Med 2018; 183(3/4), 353-363. PMID: 29635566
3. Berntson, GG, Quigley, KS, Jang, JF & Boysen, ST. An approach to artifact identification: application to heart period data. Psychophysiology 1990; 27(5), 586-598. PMID: 2274622

4. Porges, SW, Bohrer, RE, Analyses of periodic processes in psycho-physiological research. In: Cacioppo, JT, Tassinary, LG (editors.), Principles of Psychophysiology: Physical, Social, and Inferential Elements. Cambridge University Press, New York, 1990: ISBN-10: 0521348854. p. 708–753.
5. Denver, JW, Reed, SF, Porges, SW. Methodological issues in the quantification of respiratory sinus arrhythmia. Biological Psychology 2007; 74, 286–294. PMID: 17067734
6. Lewis, GF, Furman, SA, McCool, MF, & Porges, SW. Statistical strategies to quantify respiratory sinus arrhythmia: are commonly used metrics equivalent? Biological Psychology 2012; 89(2), 349-364. doi:10.1016/j.biopsycho.2011.11.009
